# Oral Health Status and Dental Care Needs Among Long-Term Care Facility Residents in Warsaw: A Cross-Sectional Study

**DOI:** 10.3390/dj14020090

**Published:** 2026-02-04

**Authors:** Julia Maria Brulińska, Aleksandra Sokołowska, Joanna Peradzyńska, Dominika Gawlak

**Affiliations:** 1 Studenckie Koło Naukowe przy Zakładzie Stomatologii Zintegrowanej, Warszawskiego Uniwersytetu Medycznego, 02-097 Warszawa, Poland; 2 Zakład Epidemiologii i Biostatyki, Wydział Lekarsko-Stomatologiczny, Warszawski Uniwersytet Medyczny, 02-106 Warszawa, Poland; 3 Zakład Stomatologii Zintegrowanej, Warszawskiego Uniwersytetu Medycznego, 02-097 Warszawa, Poland; dominika.gawlak@wum.edu.pl

**Keywords:** dental care, nursing home, oral health, aged

## Abstract

**Highlights:**

**What are the main findings?**

- The results revealed poor oral hygiene among the residents and the mean number of missing teeth ranged from 22 to 24.
- The residents had limited access to dental care, and a high prevalence of prosthetic treatment needs.

**What are the implications of the main findings?**

- The analysis emphasizes the need for structured oral health prevention programs in long-term care facilities.
- Results underscore the urgent necessity to improve access to dental services for institutionalized elderly individuals.

**Abstract:**

**Background:** Oral health is a key component of general health and quality of life in the elderly. Residents of long-term care facilities (LTCFs) are particularly vulnerable to poor oral health due to multimorbidity, polypharmacy, and dependence on caregivers. Despite increasing awareness of this issue, dental needs in institutionalized populations remain largely unmet. **Objectives:** The objective of this study was to evaluate the dental treatment needs of LTCF residents in Warsaw. The analysis focused on oral health status, oral hygiene practices, difficulties with food intake, and the need for assistance in daily oral and nutritional care. **Material and methods:** A cross-sectional study was conducted among 29 LTCF residents. Data collection included interviews on hygiene habits and dietary difficulties, followed by clinical examination assessing oral mucosa, dentition, prosthetic status, and plaque coverage (Plaque Index). Statistical analyses were performed using GraphPad Prism with Mann–Whitney U, Fisher’s exact, and Spearman’s rank correlation tests. **Results:** The median number of missing teeth ranged from 22 to 24. Active caries were found in 17 residents and periodontitis in 19. Oral hygiene was poor, with plaque covering up to 100.0% of tooth surfaces. Women had significantly more missing teeth than men (*p* = 0.0128). Difficulties with food intake were reported by 69.0% of residents. No significant associations were found between oral hygiene products use and dental or prosthetic status. **Conclusions:** This study revealed severely compromised oral health among LTCF residents. Extensive tooth loss, poor hygiene, and limited access to preventive dental care indicate the need for systematic, on-site oral health programs, caregiver training, and integration of dental services into standard geriatric care.

## 1. Introduction

Elderly care represents one of the most pressing challenges of modern society, particularly in the context of a rapidly aging population [1,2]. According to the World Health Organization (WHO), in 2020, the number of people aged over 60 years exceeded that of children under five. Moreover, it is estimated that by 2030, one in six individuals worldwide will be 60 years or older [3]. Ensuring adequate health care and maintaining well-being in this growing demographic has therefore become a global public health priority.

Long-term care facilities (LTCFs) constitute a crucial element of elderly care systems, providing not only residential accommodation but also assistance with activities of daily living and comprehensive medical and nursing support. Admission to LTCFs typically applies to individuals requiring continuous, 24 h care—such as older adults, patients with chronic diseases, and persons with physical or intellectual disabilities, including both adults and minors.

Informed consent from the resident or their legal representative is a prerequisite for admission to a long-term care facility (LTCF). Funding for institutional long-term care is typically structured as a cost-sharing model: residents may contribute up to 70.0% of their personal income, while the remaining expenses are subsidized by family members or covered by the local municipality [4]. LTCFs are mandated to ensure the provision of essential services, including nutritional support, personal hygiene, and professional nursing care [5].

Daily care is coordinated by an interdisciplinary team comprising registered nurses, health care assistants, and allied health professionals. Furthermore, LTCFs are required to provide access to primary care physicians and mental health support, such as psychologists. Access to other medical specialists—including dental care providers—is less frequent and is usually contingent upon referral from the primary care physician.

Emerging evidence highlights the substantial impact of oral health on overall health outcomes [6,7], including psychological well-being [8]. Nevertheless, oral health is frequently neglected, particularly among residents of LTCFs [8,9,10,11]. A considerable proportion of this population consists of older adults, in whom oral health problems arise from age-related changes in the stomatognathic system, compromised systemic health, and polypharmacy. A number of characteristic changes are observed in the oral cavity of older people. These include an increased susceptibility to caries, especially in the tooth roots, due to the reduced quantity and quality of saliva and difficulties in maintaining proper hygiene. At this age, there is also an impaired reactivity of the dental pulp, which makes diagnosis difficult and may mask the course of disease processes. Chronic periodontitis is also common, leading to loosening and loss of teeth, resulting in partial or complete edentulousness. Toothlessness limits chewing function, affects nutrition and reduces quality of life. A significant proportion of elderly people use dentures, which promotes the development of prosthetic stomatopathies manifested by inflammation and hypertrophy of the mucosa of the prosthetic base. With age, the risk of developing oral cancer also increases, especially in those with risk factors such as smoking and alcohol abuse.

In addition, mucosal pathologies are common in the geriatric population, including. leukoplakia, lichen planus or atrophic lesions, which require constant clinical follow-up [12]. Progressive functional decline further reduces both oral hygiene awareness and manual dexterity, exacerbating oral health deterioration. Consequently, this group presents with complex and unmet dental needs, often requiring prosthetic rehabilitation [12,13,14]. However, access to dental services in LTCFs remains limited, leading to advanced stomatognathic disorders [15,16]. Inadequate oral care, in turn, may impair quality of life; restrict nutritional intake [17,18]; and contribute to the progression of systemic conditions, including gastrointestinal, cardiovascular diseases, malnutrition, diabetes, and respiratory conditions. This highlights the importance of oral health to the overall health of older people.

## 2. Objectives

The aim of this study was to evaluate the dental treatment needs of residents in long-term care facilities in Warsaw. The analysis focused on evaluating oral health status, oral hygiene behaviors, difficulties related to food intake, and the level of assistance required for daily oral and nutritional care. Furthermore, the study sought to identify the most prevalent oral health conditions in this population and to propose potential dental care strategies aimed at improving overall oral function and quality of life.

## 3. Materials and Methods

### 3.1. Study Population

The study population consisted of residents of LTCFs in Warsaw who provided informed consent to participate. Institutional approval from the LTCF management was obtained prior to study initiation.

Exclusion criteria included a diagnosis of dementia, and lack of proficiency in Polish preventing completion of the questionnaire. Inclusion criteria included the ability to communicate verbally, the ability express informed consent in writing, and a general condition allowing for clinical examination of the patient. No age restrictions were applied, and the presence of systemic diseases did not affect eligibility.

Of the nine LTCFs invited, seven declined participation. Ultimately, 29 residents from two LTCFs were enrolled. All participants were verbally communicative, informed of the study objectives, and provided written informed consent. The study protocol was approved by the Bioethics Committee (KB/64/2024).

### 3.2. Study Design

The study incorporated both subjective and objective assessments. The questionnaire was developed specifically for this study. The clinical study was conducted simultaneously by two dentists, who were fifth-year dental students at the time and had been trained for the study by a Doctor of Medical Sciences with extensive clinical experience.

- Survey Component: Data were collected on demographic characteristics (age, sex), oral hygiene practices (frequency of toothbrushing, type of toothbrush, use of toothpaste, mouth rinses, dental floss, and irrigators), date of the last dental visit, prosthetic hygiene (cleaning methods, storage practices, nighttime removal), difficulties in food intake (a simple single-item question), and the need for assistance with oral hygiene (self-report).
- Clinical Examination: The extraoral assessment included evaluation of facial symmetry, presence of lesions, temporomandibular joint function, and masticatory muscle condition. The intraoral assessment encompassed examination of the oral mucosa, dentition status, missing teeth, and presence and condition of prosthetic appliances. Periodontal status was assessed using the Plaque Index (PI-PCR). Owing to extensive plaque deposits, periodontal pocket depth and bleeding on probing (BoP) were not evaluated. The presence of periodontitis was assessed on the basis of clinical examination, taking into account tooth loss attributable to periodontal disease, pathological tooth mobility, visible loss of periodontal tissues, and the presence of exposed furcations. In addition, typical inflammatory features such as gingival redness, swelling, and bleeding were considered.

All participants received individualized oral hygiene instructions. Dental treatment needs were categorized by specialty: oral surgery, endodontics, restorative dentistry, prosthodontics, and periodontology.

### 3.3. Statistical Analysis

Data were analyzed using GraphPad Prism version 10.5 software. Continuous variables were expressed as medians, interquartile ranges, and minimum–maximum values. Due to the small sample size, non-parametric tests were applied:

- Mann–Whitney U test for quantitative variables,
- Chi-square test or Fisher’s exact test for categorical variables,
- Spearman’s rank correlation coefficient to assess relationship between quantitative or ordinal variables.

A *p*-value < 0.05 was considered statistically significant. The study was conducted in accordance with the principles of the Declaration of Helsinki, ensuring anonymity and voluntary participation.

## 4. Results

The study included 29 residents of LTCFs in Warsaw. Based on their level of independence in performing oral hygiene, participants were divided into two groups: independent (*n* = 25) and dependent (*n* = 4). The groups were markedly unequal in size, which should be considered when interpreting the results.

The mean number of missing teeth, including third molars, was higher among dependent participants (25.0 ± 8.1) compared with independent residents (21.0 ± 7.8). However, this difference did not reach statistical significance (*Mann–Whitney U* = 35.0, *p* = 0.3562).

### 4.1. General Oral Health Status

Overall, the oral health of the studied population was poor. The median number of missing teeth ranged from 22 to 24. In many cases, extensive plaque accumulation was noted, affecting up to 100.0% of tooth surfaces.

Oral hygiene practices were inadequate. The average brushing frequency was 1.33 times per day, with a median of once daily, and only 63.6% of residents reported using fluoride toothpaste. Night-time denture removal was practiced by 62.5% of denture wearers, and 50.0% stored their dentures in a dedicated container.

Dependence in performing oral hygiene was not significantly associated with plaque levels; however, the dependent group was very small (*n* = 2), which limited the power of statistical analysis.

### 4.2. Comparison of Oral Pathologies

The prevalence of selected oral pathologies was compared between the two groups using Fisher’s exact test. Active carious lesions were diagnosed in 16 independent and 1 dependent participant (*p* = 0.2677). Gingivitis was identified in 21 independent and 2 dependent participants (*p* = 0.3188), whereas periodontitis was observed in 19 and 2 participants, respectively (*p* = 0.4217). None of these differences were statistically significant.

Similarly, no significant differences were observed with respect to prosthetic status, or the number of teeth indicated for extraction. Partial removable dentures were used by 20.0% of independent and 25.0% of dependent participants, while complete dentures were worn by 28.0% and 25.0% of these groups, respectively. The mean number of teeth indicated for extraction was 0.60 in the independent group and 0.33 in the dependent group, and comparisons using Fisher’s exact test and mean analyses confirmed the absence of statistically significant differences. Prevalence of different types of dentition and prosthetic restorations among dependent and independent participants is shown in Table 1.

### 4.3. Association Between Age and Dental Status

Age did not have a statistically significant impact on dental status. The median age of edentulous residents (*n* = 6) was 88 years, compared with 84 years among participants with remaining dentition (*n* = 23). This difference was not statistically significant (*Mann–Whitney U test*, *p* = 0.6858). Similarly, Spearman’s rank correlation revealed no significant association between age and the number of missing teeth (*r* = 0.141, *p* = 0.4667).

### 4.4. Associations Between Dentition Status and Eating Difficulties

Among all participants, 20 residents (69.0%) reported difficulties with food intake, regardless of food consistency. Comparisons between residents with eating difficulties (*n* = 20) and those without such problems (*n* = 9) revealed that complete natural dentition was present only among participants without eating difficulties (11.1% vs. 0.0%, *p* = 0.3103). Complete dentures were more common in those experiencing difficulties (35.0% vs. 11.1%, *p* = 0.3715), as were fixed prosthetic restorations (35.0% vs. 11.1%, *p* = 0.3715).

No statistically significant differences were found in the frequency of partial removable dentures (20.0% vs. 22.2%, *p* = 1.0000) or skeletal (frame) dentures (10.0% vs. 11.1%, *p* = 1.0000). Although these differences were not statistically significant, the observed trend suggests that residents reporting eating difficulties were more likely to depend on prosthetic restorations, reflecting poorer natural dentition.

### 4.5. Plaque Assessment

The percentage of plaque-covered surfaces (PI–PCR) was analyzed. The median PI–PCR value in both the independent (*n* = 21) and dependent (*n* = 2) groups was 100.0%. The Mann–Whitney U test did not demonstrate statistically significant differences between the groups (*U* = 19, *p* > 0.9999). The difference in medians was 0.0 percentage points (95.26% CI: 0.0 to 20.0). These findings should be interpreted with caution due to the very small sample size in the dependent group.

### 4.6. Associations Between Oral Hygiene Practices and Tooth Loss

The relationship between oral hygiene behaviors and the number of missing teeth was also analyzed. Residents who used fluoride toothpaste had, on average, 20.5 missing teeth, compared with 22.2 among those who did not (*p* = 0.684). Participants who used mouth rinses had a mean of 20.2 missing teeth, compared with 21.5 among non-users (*p* = 0.910). The use of a manual toothbrush was associated with a mean of 19.8 missing teeth, whereas those using other cleaning methods had an average of 23.6 (*p* = 0.267).

Residents who practiced night-time denture removal had a mean of 22.4 missing teeth, compared with 27.5 among those who did not (*p* = 0.140). Finally, participants with dietary restrictions had a mean of 23.0 missing teeth, compared with 21.2 among those without such restrictions (*p* = 0.551).

All comparisons were performed using the Mann–Whitney U test, and none of the differences reached statistical significance. However, a trend toward fewer missing teeth was observed among participants adhering to standard oral hygiene practices, particularly those using fluoride toothpaste, mouth rinses, and manual toothbrushes. Correlations between oral hygiene practices and the average number of missing teeth among residents of long-term care facilities are shown in Table 2.

### 4.7. Correlation Between Oral Hygiene Practices and Prosthetic Status

The association between oral hygiene behaviors and prosthetic status was evaluated using Fisher’s exact test. No statistically significant relationships were identified between the use of hygiene measures (fluoride toothpaste, mouth rinses, dental floss, manual toothbrushes, night-time denture removal, or dietary restrictions) and the presence of natural dentition, partial removable dentures, complete dentures, skeletal (frame) dentures, or fixed prosthetic restorations (all *p* > 0.05).

### 4.8. Dental Care Utilization

The availability and type of dental care were also assessed. For 22 participants, information regarding the time since the last dental visit was available. Spearman’s rank correlation demonstrated a weak, non-significant positive association between the number of missing teeth and time since the last dental visit (*r* = 0.302, *p* = 0.1721).

Residents whose most recent dental visit was pain-driven (*n* = 6) had a higher median number of missing teeth (23.5) compared with those attending for preventive check-ups (*n* = 13; median = 17.0). This difference approached statistical significance (*Mann–Whitney U test*, *p* = 0.0527), suggesting that an interventional rather than preventive pattern of dental attendance may be associated with poorer dental status.

### 4.9. Association Between Sex and Dental Condition

**Figure 1:**
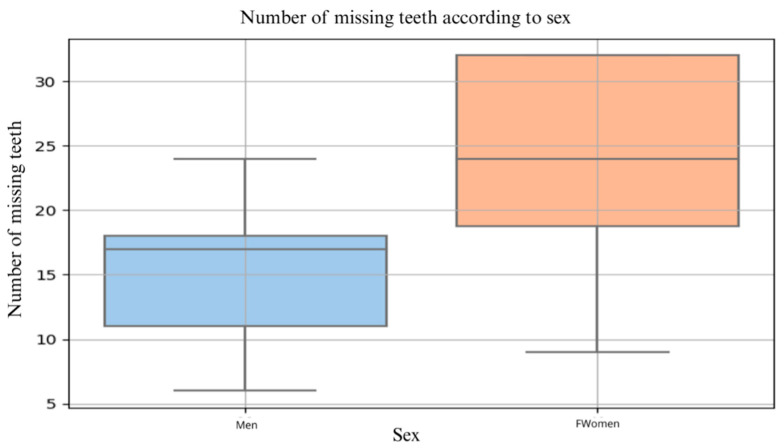
Distribution of missing teeth according to sex among LTCF residents. Median number of missing teeth: women—24; men—17 (Mann–Whitney U test, *p* = 0.0128).

Sex was significantly associated with dental condition. Among the 29 participants, 20 were women and 9 were men. Women had a significantly higher number of missing teeth compared with men, with median values of 24 and 17, respectively (*Mann–Whitney U test*, *p* = 0.0128). Distribution of missing teeth according to sex among LTCF residents is illustrated in Figure 1.

## 5. Discussion

### 5.1. General Condition of the Dentition

The present study demonstrated that the overall oral health of LTCF residents in Warsaw was poor. The median number of missing teeth was high (22–24). Active carious lesions were found in 17 out of 29 participants, and periodontitis in 19. Nearly 70.0% of respondents reported difficulties with food intake. Importantly, all residents required some form of dental intervention, including restorative, surgical, prosthetic, or periodontal treatment, as well as improvement in oral hygiene practices.

These findings are consistent with previous research in similar populations. A study conducted by the Medical University of Łódź found that 46.0% of LTCF residents were edentulous, while nearly 60.0% required dental intervention [10]. In a study by Janssens et al. from Belgium, edentulism was reported in 41.9% of residents, and 77.0% required dental treatment [19].

The present results highlight the unsatisfactory oral health status of LTCF residents and underline the need for systematic improvement. Oral health directly influences general health: periodontitis has been linked to an increased risk of atherosclerosis and diabetes mellitus [20], oral diseases contribute to respiratory disorders, and poor hygiene increases the risk of dementia [20]. Additionally, the condition and functionality of the dentition significantly affect mental health. A study from the Pomeranian Medical University found that 79.3% of LTCF residents experienced depressive symptoms, and a positive correlation existed between impaired masticatory function and depression [8].

In Poland, increasing attention is being paid to the importance of oral health in older adults and its impact on general health. In 2020, recommendations for older adults, LTCF residents, and their caregivers were issued by the Polish Branch of the Alliance for a Cavity-Free Future (ACFF) [21]. Despite growing awareness among physicians, dentists, and long-term care staff, the oral health and hygiene of LTCF residents remain unsatisfactory.

An interesting finding of the present study was that women had a significantly higher number of missing teeth than men, a result consistent with previous reports [10].

### 5.2. Awareness Among Residents

The awareness and knowledge of residents regarding oral hygiene were unsatisfactory. The median toothbrushing frequency was once daily, and nearly one-third of participants reported brushing less than once a day or not at all. Similar findings were reported in Łódź, where only 34.7% of LTCF residents declared brushing their teeth at least twice daily [10].

The attitudes of LTCF residents toward oral health were investigated in a Belgian study [9]. Interviews concerning daily hygiene routines and perceptions of professional dental care revealed that many residents had not visited a dentist for several years, often since admission to the facility. Most expressed no intention of seeking future dental care [9]. Many were unaware of the relationship between oral and systemic health and lacked knowledge of proper denture hygiene, although they recognized the influence of oral health on chewing and speech. The authors also emphasized physical limitations that hinder self-care and barriers to accessibility, especially for wheelchair users, when accessing dental offices [9].

### 5.3. Access to Dental Care and Frequency of Visits

The results also suggest a potential relationship between limited access to dental care and poor oral health status. Although the correlation between time since the last dental visit and the number of missing teeth did not reach statistical significance, a trend was observed indicating that residents who visited the dentist less frequently had a higher number of missing teeth.

Furthermore, participants whose last dental visit was pain-driven had a markedly higher median number of missing teeth compared with those who attended for preventive check-ups. This observation aligns with findings from a Saarland study, in which systematic dental care was rare, and most visits were emergency-based [16].

These data also indicate that the reason for the last dental visit may play a significant role in oral health outcomes. The pattern observed—more missing teeth among those attending for pain rather than prevention—underscores the importance of preventive dental care. Comparison with the Saarland study suggests a systemic issue: the absence of organized, regular dental care programs in LTCFs contributes to oral health deterioration [16]. Therefore, ensuring accessible and preventive dental services for LTCF residents is essential to improving oral health outcomes and reducing tooth loss. Regular visits by dentists, dental hygienists and specialists, or on-site preventive campaigns, could enable early detection and treatment of emerging problems before systemic and general dental problems arise from untreated oral pathologies. We recommend establishing structured partnerships between care facilities and local dental services, a contract with the National Health Service or Medical Universities with Dental Departments, as such arrangements can improve the coordination of planned interventions, strengthen continuity of care, and promote the preservation of functional dentition and overall oral well-being among residents.

### 5.4. Support and Awareness of Caregivers

Caregiver knowledge and involvement in oral hygiene also appeared to be insufficient. Although no significant differences were found in plaque accumulation between independent and dependent residents, this was most likely due to generally poor hygiene across both groups rather than to effective caregiver support. The median PI–PCR score (percentage of plaque-covered tooth surfaces) in both groups was 100.0%, confirming widespread plaque accumulation.

A similar study conducted in Łódź found that 47.2% of LTCF residents presented with poor oral hygiene [10]. The same study reported that 25.9% of participants recognized their reduced ability to maintain oral hygiene, and many of those who requested assistance did not receive it [10]. In Sweden, 71.5–87.1% of LTCF residents required assistance with oral hygiene procedures, yet only 6.9% received such support [22]. The importance of maintaining proper oral hygiene is further emphasized by the Polish Patient Ombudsman, who published two sets of recommendations for long-term care facilities on the official website [23].

Interviews with employees of the LTCFs included in this study revealed a lack of knowledge about oral health. Surprisingly, many staff members were unfamiliar with even basic recommendations. Although this observation was not included in the formal study analysis, it highlights the need for further research. We believe that training programs for LTCF personnel focused on the fundamentals of oral health care are essential.

The effectiveness of such educational interventions has been confirmed in a Swedish study, in which the oral health knowledge of LTCF employees improved significantly following training sessions conducted over six months [24]. These findings support the recommendation for systematic, recurring staff education in oral health care for long-term care facilities.

### 5.5. Nutritional Difficulties—Patient Health

Nutritional difficulties were reported by most participants. Although no statistically significant association was found between eating difficulties and prosthetic status, the observed trends suggested that residents experiencing such problems were more likely to use complete dentures or fixed prosthetic restorations, whereas natural dentition was observed only among those without reported difficulties. These findings are consistent with the observations of Natapov et al. from the Mabat Zahav (National Health and Nutrition Survey of the Elderly in Israel), which demonstrated that individuals with tooth loss, prosthetic rehabilitation, or impaired mastication had poorer dietary intake, including reduced consumption of energy, protein, fiber, and vegetables [17]. A clear association has been established between tooth status, regular dental attendance, diet quality, and overall health [17]. It has been observed that individuals wearing complete dentures are more likely to experience difficulties with chewing. Despite their rehabilitative role, prosthetic restorations—particularly complete dentures—cannot fully restore masticatory efficiency to the level of natural dentition [25]. Missing teeth or ill-fitting dentures may impair eating comfort, limit food selection, and increase the risk of nutritional deficiencies. Maintaining at least 20 natural or artificial teeth is considered the threshold necessary for adequate masticatory function [26]. None of the participants reporting eating difficulties met this criterion, which may explain their self-reported problems. The findings of the present study, together with those of the Mabat Zahav project, emphasize the importance of preserving natural dentition whenever possible. In cases of tooth loss, they highlight the need for well-fitted prosthetic restorations and regular dental follow-up for older adults. Improving the availability and accessibility of prosthetic and preventive dental care—particularly within LTCFs—may enhance quality of life and help prevent malnutrition and its systemic consequences.

### 5.6. Study Limitations

This pilot study has several limitations. The sample size was small (*n* = 29), particularly in the group of dependent residents, which reduced the statistical power of the analyses. Information regarding systemic comorbidities and general health status was not collected, which limited the ability to assess potential confounding factors. In addition, communication difficulties with some participants prevented complete data acquisition.

A limitation of this study was the inability to measure periodontal pocket depth (PD) and bleeding on probing (BoP) due to the presence of abundant soft and hard deposits. The assessment of these parameters was abandoned to avoid the risk of exacerbating bacterial infection and additional pocket infection. Consequently, these indicators were excluded from the study form, even though they were originally planned to be used to determine the stage of periodontitis. For this reason, the analysis only considered the presence of inflammation, without the possibility of classifying its clinical stage.

Another limitation concerns the descriptive nature of the study, which did not allow for standardized diagnostic classification in the data collection forms (e.g., specific types of oral mucosal lesions or prosthetic restorations). Furthermore, cognitive impairment and signs of senile dementia, observed in several participants, hindered the accurate recording of subjective data. Finally, the observational cross-sectional design of the study precludes any causal inferences regarding the relationships identified.

## 6. Conclusions

This study provides a consolidated synthesis of evidence indicating that residents of long-term care facilities face significant and consistently unmet oral health needs. The deficits observed—including inadequate support for daily oral hygiene, limited access to professional dental care, and inadequate denture use and hygiene—collectively point to a structural inadequacy in the current provision of oral health care in institutional settings. These findings identify a clear pattern in which suboptimal practices contribute directly to progressive deterioration in oral function and comfort.

Placed within the broader context of geriatric care, these findings reinforce the need to implement rigorously structured oral hygiene programs. Regular on-site dental examinations and prosthetic assessments should be institutionalized as standard practice, given their role in preventing advanced disease and maintaining oral function. Equally important is the systematic education of both staff and residents in evidence-based preventive strategies that can ensure lasting improvements in oral health outcomes and reduce the overall need for invasive treatment.

Education of both staff and residents in preventive oral health practices should be prioritized. Integrating dental care into the standard framework of geriatric and institutional care—both organizationally and financially—would help ensure consistent, accessible, and comprehensive dental services.

## Figures and Tables

**Table 1:** Prevalence of different types of dentition and prosthetic restorations among dependent and independent participants.

	Presence in Dependent Individuals (%)	Presence in Independent Individuals (%)	*p*-Value
Complete dentition	0.0	11.1	0.3103
Partial denture	20.0	22.2	1.0
Full denture	35.0	11.1	0.3715
Frame prosthesis	10.0	11.1	1.0
Fixed prosthesis	35.0	11.1	0.3715

**Table 2:** Association between oral hygiene practices and the average number of missing teeth among LTCF residents.

Hygienic Variable	Average Number of Missing Teeth (Using Individuals)	Average Number of Missing Teeth (Not Using Individuals)	*p*-Value
Use of fluoride toothpaste	20.5	22.2	0.684
Use of mouthwash	20.2	21.5	0.910
Use of a manual toothbrush	19.8	23.6	0.267
Implementation of night breaks	22.4	27.5	0.140
Dietary restrictions	23.0	21.2	0.551

## Data Availability

The datasets generated and analyzed during the current study are available from the corresponding author on reasonable request.
